# The Congress of American Physicians and Surgeons

**Published:** 1888-10

**Authors:** 


### THE CONGRESS OF AMERICAN PHYSICIANS AND SURGEONS.

The first of what is contemplated as a triennial congress of physicians and surgeons, to be held in Washington, occurred in that city on September 17, 18, and 19. In accordance with the announcement previously made, the congress was composed of most of the National societies of the various special departments of the medical profession, which held separate sessions of those societies during the day, and met in general congress during the evening. The attendance, whilst not very large, was fairly representative of the better element of the medical profession of our country, and a number of prominent physicians and surgeons from Europe were present, and participated in the work of the congress, and of the various societies.

The scientific work, whilst it was not all of equal merit, was, both in the congress and in the societies, of such a character as to be a fair index of the most advanced ideas of medical and surgical science of the day.

So many societies being in session at the same time made it an exceptionally difficult occasion for securing accurate reports of the work done in the different societies, but from the report of the representative of the *Journal and Examiner*, and from those of some of the weekly journals, we are enabled to give in this issue an epitome of the work of the congress, and of societies composing the congress, and the by-laws which are to govern the next congress.

Whilst this one adds one more to the multiplicity of medical organizations in this country, it will have an advantage over all of the others in the fact that it is to occur but once in three years. If some of the other organizations should not call their members together more frequently than every three years, it might prove a boon to the medical profession, even if there should prove to be fewer offices to be sought.

